# Potentially preventable premature deaths in women and men from the two leading causes of death in Austria, mortality statistics of the nine federal states 2010–2012

**DOI:** 10.1186/s12889-015-2502-y

**Published:** 2015-11-25

**Authors:** Éva Rásky, Erwin Stolz, Nathalie Tatjana Burkert, Franziska Großschädl

**Affiliations:** Institute of Social Medicine and Epidemiology, Medical University of Graz, 8010 Graz, Austria

**Keywords:** Mortality statistics, Preventable death, Regional differences, Sex-specifity, Diseases of the circulatory system, Cancer, Austria

## Abstract

**Background:**

In Austria, mortality from diseases of the circulatory system and malignant neoplasms is high and varies among the federal states. Lower mortality in some states indicates a preventive potential in those states with higher mortality.

**Methods:**

We computed the number of premature deaths, for women and men separately, from the two leading causes of death, diseases of the circulatory system (ICD-10: I00-I09) and cancer (ICD-10: C00-C97), in the nine Austrian federal states between 2010-2012. The potentially preventable deaths per federal state and sex were calculated by subtracting expected deaths from observed deaths.

**Results:**

The western federal states had the lowest death rates, and thus the smallest preventive potential. In death from circulatory diseases and from cancer the differences between women and men varied remarkably between the federal states. For circulatory diseases among all federal states the highest difference in percent was given in Vorarlberg (6.2 %) with more potentially preventable deaths for men. For cancer, Burgenland had the highest difference (8.6 %) in comparison with the other federal states, again with the higher preventive potential for men.

**Conclusions:**

Intervention programs as lifestyle modification interventions as well as improvements in health care services provision, should focus on the characteristics of the specific federal state, which are setting-oriented and account for social determinants including sex/gender differences and economic factors. Relevant data gathering is therefore, urgently needed.

## Background

As in other industrialized countries, life expectancy in Austria has been rising to an unprecedented level. For women, life expectancy at birth on average amounts to 83.3 years, and to 78.0 years for men [1]. The two leading causes of death between 2010 and 2012 were identical for women and men. Diseases of the circulatory system and malignant neoplasms together caused 68.2 % of all deaths in 2013 [2]. However, in the last decade, the age-standardized rate of mortality due to diseases of the circulatory system decreased by 30.4 % [2]. Ford et al. [3] showed in their systematic review that approximately 44 % of the decline of the coronary heart disease mortality in the United States between 1980 and 2000 was attributable to changes in major risk factors as well as to cardiac treatment interventions. These findings matched those from other industrialized countries [4–6].

In Austria, the mortality due to malignant neoplasms also decreased by 9.4 % in the last decade even though cancer incidence itself had increased within the same time period [2]. In regard to cancer mortality, experts observe that better prevention, screening, and treatment are keys to this continuous progress [7]. The most significant modifiable risk factors for diseases of the circulatory system and of cancer are nutrition, physical activity, and smoking [8, 9]. The World Health Organization [10] states that over three-quarters of the cardiovascular mortality may be prevented by adequate changes in lifestyle. Risk reduction, especially smoking cessation, could prevent 40 % of the cancer incidence [11]. Although these lifestyle-related risk factors vary in their significance for the two leading causes of deaths in Austria, prevention could be an important measure to decrease specific mortality and, therefore overall mortality. Applying the article of Yoon et al. [12] as a model, we analyzed sex-specific mortality data on the two leading causes of death to determine potentially preventable, premature, deaths in Austria. In general, and in each of the nine federal states of Austria, the two leading causes of death are identical for women and men. However, the rates of death from each of these causes vary across the federal states and between sexes. We therefore assume that lower sex-specific mortality in certain states indicates a preventive potential in those federal states presenting higher sex-specific mortality. Previous research documented a geographic east–west gradient in mortality in European countries [13, 14]. In Austria, cardiovascular mortality [15, 16] is probably linked to regional socioeconomic disparities between the wealthier western and poorer eastern federal states, with the exception of the capital city and federal state Vienna in the east of Austria [17].

Existing differences in death rates in Austria stratified by region and sex could identify the need for implementing preventive programs to reduce mortality, including more effective health care services provision, in specified federal states, and specifically targeting women or men.

## Methods

The national vital statistics mortality data from the Austrian statistical office is based on death certificates, including the information on the last residence of the deceased, and corresponds with international standards in data collection and publication complying with the WHO guidelines (ICD-10) and EC Regulation 328/2011. We used mortality statistics and population estimates of 2010–2012, for women and men separately, to compute the number of deaths for the two leading causes of death, diseases of the circulatory system (ICD-10: I00-I09) and malignant neoplasms (ICD-10: C00-C97) [18], in the nine federal states. The calculation followed the procedure outlined by Yoon et al. [12], however, augmented by the variable sex. The number of annual potentially preventable, premature, deaths, i.e. death before age 83 for women and age 78 for men, was determined by comparing the annual death rate of the federal state with the lowest rate for both causes of death between 2010–2012 with the number of the observed deaths in the eight other federal states during the same time period.

The number of potentially preventable deaths for each cause of death was calculated in three steps. At first, we calculated disease-specific annual death rates by age groups (0–49, 50–59, 60–69 and 70+ years) in order to control for age differences, for women and men separately, in the federal state populations. Then we ranked the federal states accordingly. The state with the lowest death rate for cause of death, sex and age-group per year, was selected as the corresponding reference value. Secondly, based on this benchmark, the number of expected deaths for each age and sex group was computed and summarized. Finally, the premature deaths for the two leading causes of death per federal state were calculated by subtracting expected deaths from the observed deaths-the average of the annual results from 2010–2012-for women and men respectively.

Age standardization across four age groups (0–49, 50–59, 60–69, 0+) represents the lowest age-stratification possible while ensuring a sufficient number of cases per state, year, cause of death and sex. The age group “0–49” was not differentiated further, due to low number of deceased. The age-group “70+” was not disaggregated further, because the different cut-off points for men and for women due to differing average life expectancies (78 years for men, 83 for women) would have led to unequal numbers, and therefore to less comparable age groups. In addition, the proportion of the age-group “70+” for men (range = 6.0–8.2 %) and women (range = 10.5–13.9 %) was comparable across the federal states. Nonetheless, this aggregation of deaths over this wide age interval could have caused sub-optimal age-standardization.

## Results

The calculated average death rates per 100,000 population of the federal states and sex between 2010 and 2012 were in general lowest in the western states of Austria. For this time period, we found the lowest death rates for cancer in Salzburg for both men (1,612) and women (1,158), for cardiovascular diseases in Tyrol for men (1,244), and for women in Vorarlberg (957). Hence, the western federal states Salzburg, Tyrol, and Vorarlberg had the least potential of preventable premature deaths in the two leading causes of death among women and among men (see Tables 1 and 2). While potentially preventable deaths in women and in men from diseases of the circulatory system and cancer amount to less than 15 % in the western states, these two causes of death increase to more than 30 % in Vienna.

**Table 1:** Deaths and potentially preventable deaths (absolute numbers and percentage) for the two leading causes of death in the nine federal states, Austria, stratified by sex, 2010-2012

Diseases of the circulatory system (I00–I99)						
Federal states	Deaths observed (n)		Potentially preventable deaths (n)		Potentially preventable deaths (%)	
	Males	Females	Males	Females	Males	Females
Vorarlberg	197	185	21	9	10.9	4.7
Tyrol	355	379	11	30	3.0	7.9
Salzburg	298	308	40	41	13.3	13.4
Carinthia	375	434	76	104	20.2	23.9
Styria	861	896	227	197	26.4	22.0
Upper Austria	928	1 008	223	257	24.0	25.4
Lower Austria	1 174	1 281	293	368	25.0	28.7
Burgenland	232	266	69	88	29.6	33.1
Vienna	1 231	1296	447	445	36.3	34.4
Total	5 652	6 052	1 407	1 539	21.0	21.5

**Table 2:** Deaths and potentially preventable deaths (absolute numbers and percentage) for cancer in the nine federal states, Austria, stratified by sex, 2010–2012

Cancer (C00-C97)						
Federal state	Deaths observed (n)		Potentially preventable deaths (n)		Potentially preventable deaths (%)	
	Males	Females	Males	Females	Males	Females
Vorarlberg	271	247	34	33	12.7	13.2
Tyrol	517	492	53	69	10.2	14.0
Salzburg	366	367	16	40	4.5	10.9
Carinthia	493	484	91	99	18.5	20.4
Styria	1 047	1 015	200	204	19.1	20.1
Upper Austria	1 147	1 046	201	162	17.6	15.5
Lower Austria	1 482	1 360	306	292	20.7	21.5
Burgenland	288	240	70	38	24.2	15.6
Vienna	1 522	1 566	458	521	30.1	33.3
Total	7 133	6 816	1 430	1 457	17.5	18.3

When focussing on premature deaths for diseases of the circulatory system and cancer in Austria in total separately, the percentages in potentially preventable deaths hardly differ between women and men. Interestingly, when the focus is turned on single federal states, the sex-specific difference in percentages varies remarkably. In the case of circulatory diseases, these differences amount to more than three percent in five of the nine federal states.

The prevention potential in Vorarlberg, Styria and Vienna is higher for men than for women. Focussing in the case of cancer on sex-specific potentially preventable deaths the differences between women and men amount to nearly nine percent, four federal states differ in more than three percent. The prevention potential is higher for women in Tyrol, Salzburg and Vienna than in the other federal states.

## Discussion

Socioeconomic conditions, biological and psychosocial factors as well as health care service provision, prevention programs as well as individual lifestyles are major determinants for health and illness of women and men. Diseases causing a great number of deaths and prematurely occurring deaths in a particular population indicate a need for preventive interventions on all these determinants. The World Health Organization points out that setting-oriented interventions enhance the effectiveness and sustainability of such programs [19, 20]. In Austria, more than two thirds of deaths between 2010 and 2012 were caused either by diseases of the circulatory system or by cancer. Comparing those Austrian federal states with high death rates with those with low rates due to the two leading causes of death shows that up to one third of these deaths are potentially preventable. These results are consistent with the 20 % to 40 % of premature preventable deaths analysed by Yoon et al. [12] for the United States.

These findings suggest that the preventive potential for diseases of the circulatory system as well as for cancer needs to be realized. Particularly the eastern and southern federal states of Austria-Burgenland, Vienna, Styria and Carinthia -have a high potential for reducing premature mortality. In some federal states, the differences between women and men are particularly striking. The potentially preventable deaths due to circulatory diseases in Tyrol are 7.9 % for women and only 3.0 % for men, whereas for cancer the percentage in Burgenland is 15.6 % for women, and 24.2 % for men (see Tables 1 and 2). For circulatory diseases these differences are particularly high in Vorarlberg and Styria favouring women with lower preventive potentials for deaths in both federal states. Women in Salzburg had the highest rate of potentially preventable deaths from cancer. In Burgenland men had the highest rate of potentially preventable deaths from cancer. It is highly relevant to know about these sex-specific disparities in potentially preventable deaths in Austria to open the floor for further research on the causal effects, as their causes are not yet clear.

Economic data reveal disparities between the populations in south-eastern and western Austria. Median income from employment differs between women and men, and among the federal states [21]. This gap is at least to some extent likely to explain the differences in mortality between the federal states. In addition, the distribution of unhealthy lifestyle factors varies between the states. Long-term trends show regional lifestyle-differences clearly, with healthier lifestyles in the west of Austria, both in women and men [22, 23]. In terms of health care services provision, women with cerebrovascular diseases in Styria, for example, attain insufficient therapeutic interventions which could also be a reason for premature deaths in women [24]. Although such regional disparities are plausible co-factors for the differences assessed, analyses of aggregate-level data does not allow to distinguish between individual-level factors, composite measures of variables and context effects on regional mortality. A further limitation of the study is using the current national life expectancy as an age limit for preventable deaths. It could exclude some preventable deaths due to cardiovascular disease and cancer if life expectancy is reduced by higher mortality due to other causes. In addition, our analyses are based on single causes of death reported in death certificates. These might be affected by reporting bias. Biased regional differences, however, seem unlikely, as the autopsy rate, an indicator for confirmed causes of deaths, shows. The mean autopsy rate in Austria between 2010 and 2012 was on average 15.7 % of all deceased [25]. In this period, Carinthia and Styria had rates below 10 %, Salzburg almost 12 %, Burgenland and Tyrol almost 15 %, and the other federal states were above the mean average. Quality checks for autopsies and for non-autopsy deaths are non-existent in Austria. Sex-specific numbers of autopsies are also not available in Austria, additionally limiting sex-specific analyses.

Finally, and in contrast to the study by Yoon et al. [12] on 50 states, our study encompasses the nine Austrian federal states. Therefore, just one federal state annually was taken as a benchmark for computing preventable deaths. The minimum death rates were averaged across the three years of study and stratified by age-group, sex, and cause of death and yielded robust results as 14 of the 16 computed minimum death rates were located in the three western states of Austria (Vorarlberg, Tyrol, Salzburg). Therefore, we consider this a reasonable procedure.

## Conclusions

Mortality is dependent on social determinants. As public health experts we therefore advise policy makers to focus on improving general education in Austria, implementing public employment schemes and intensifying programs for sex- and gender- specific prevention, health promotion and health care provision, especially in the eastern federal states having a higher potentially preventable mortality [26]. Disease- and sex-/gender-specific approaches are more effective than general media campaigning usually practised in Austria [27, 28]. During the past years, Austria did initiate health promotion, enhance health literacy in the general population, a health care reform, and a cancer control plan, but these strategies have yet to be realized. In addition, sex-/gender-specific strategies are absent in most of these programs and largely in health care services provision [29], although strategies and tools are available [30, 31]. Target group-specific preventive programs are particularly necessary when focussing on regional differences [32]. Failure to reduce this unequally distributed mortality will be detrimental both to women and men as well as to all of society.
